# Easy on-demand single-pass self-assembly and modification to fabricate gold@graphene-based anti-inflammatory nanoplatforms

**DOI:** 10.1038/srep34890

**Published:** 2016-10-06

**Authors:** Jeong Hoon Byeon, Jae Hong Park

**Affiliations:** 1 School of Mechanical Engineering, Yeungnam University, Gyeongsan 38541, Republic of Korea; 2 School of Health Sciences, Purdue University, IN 47907, United States

## Abstract

Zwitterionic chitosan (ZC) was modified by fully (both for lateral dimension and thickness) nanodimensional gold-graphene oxide (Au@GO) flakes under visible light and the potential of the resulting materials as biomedical nanoplatforms was investigated. Fully nanodimensional GO flakes floating in nitrogen gas were incorporated with Au nanoparticles to form Au@GO nanoflakes, and the Au@GO was then incorporated with ZC droplets to form the Au@GO-ZC hybrid nanoparticles. The collected particles were exposed to visible light to induce the photocatalytic activity of the Au@GO nanoflakes towards the ZC derivatives. The visible-light-exposed particles show different chemical and surface properties from the unexposed particles, while there were no significant differences in cytotoxicity and macrophage inflammatory protein production. This work suggests that incorporating fully nanodimensional Au@GO flakes with ZC is a suitable technique for ambient photo-modification of the chitosans’ surface property without significant changes in size and shape and increases in cytotoxicity and inflammatory response.

With the rapid development of nanotechnology and extensive applications of nanoparticles, well-designed multifunctional nanoparticles can have immense potential in biomedical applications such as imaging and therapeutics^1^. Numerous approaches have been developed for the fabrication of hybrid nanomaterials consisting of organic and inorganic components with desired sizes, shapes, and physicochemical and optical properties for efficient use as alternative materials and systems in various technological fields, such as energy, biomedicine, and micro/nanoelectronics^2^,^3^,^4^. In this regard, finding versatile, tunable and efficient strategies to prepare well-organized nanostructures with functionalities is a very important issue for current and future materials technology. The development of suitable incorporation techniques to prepare nanoscale hybrid systems is a critical issue in nanoscience and nanoengineering^5^. Particularly interesting core materials are inorganic nanoparticles, which are already used in therapeutic and imaging applications^1^,^6^,^7^. Specifically, the nanoparticles are not only employed as tracers for imaging when they are injected into the body, but also as phototransducers to generate heat to kill cancer cells by hyperthermia^7^.

In recent years, there has been huge interest in employing 2D carbonaceous materials known as “graphene” and relevant systems for biomedical applications (e.g. molecular imaging, drug delivery, and chemo- and photo-thermal therapy) since it has outstanding properties regarding structure flexibility and intensity, biocompatibility, and large surface area^8^,^9^. Owing to lots of functional groups on the surface that are available to be conjugated with other components, graphene oxide (GO) flake-polymer hybrid structures can be effectively incorporated with therapeutic materials, such as DNA, to form therapeutic nanocarriers, which exhibited good DNA delivery performance in HeLa cells^8^. This may support and extend the use of other inorganic nanomaterials, such as gold (Au), i.e., Au@GO^1^, and recently generates a new class of functional materials with improved properties and thus provides new opportunities for biomedical applications^10^. However, performance of Au@GO in biological environments is still largely unknown, particularly with regard to cellular response to GO. In particular, there are conflicting results on its inflammatory responses partially due to large variations in physicochemical properties of GO^11^,^12^. Thus, combinations of Au@GO flakes and polymers (for reducing toxicological/inflammatory responses) could have multifunctional properties for practical biomedical purposes. As far as we know, moreover, the fabrication of fully nanoscale (i.e., lateral nano-dimensional) Au@GO-polymer hybrid structures for biomedical purposes has not been yet reported since the graphene materials used so far have come from micron-sized graphite powders.

Many formulations of inorganic-organic hybrid systems based on multistep wet chemistry for biomedical applications are introduced as suspensions of solid particles and may only be workable with the initial performance for a short period of time. Moreover, organic or polymeric components incorporated with inorganic nanoparticles are normally unstable owing to gradual degradation by hydrolysis; hybrid nanomaterials in a suspension or colloidal form would therefore not be recommended^13^,^14^. As a result, the paradigm shift in the preparation strategy towards simpler, more efficient, and versatile processing to prepare stable hybrid nanomaterials for various biomedical applications makes this research area currently challenging. Gas-phase processing is one alternative that has fewer preparation steps for producing the required nanomaterials and could allow long-term storage of the resultant nanomaterials^7^. Employing gas-phase processing further enhances the process continuity in production, implying that only simple mechanical collection of materials is required without producing much waste^15^. However, conventional gas-phase synthesis of nanomaterials is commonly performed at high-temperature conditions (over 500 °C at the very least) and thus it would only be workable to synthesize inorganic or hard nanoparticles^16^. On this account, gas-phase processing to produce inorganic parts for hybrid nanomaterials in a single-pass configuration would not be suitable in the absence of post-treatment or post-functionalization steps^3^. Therefore, entirely low-temperature processing is strongly required since temperatures over 300 °C can decompose most organic materials (i.e., biofunctional soft materials)^17^.

This work introduces a novel strategy to fabricate fully nanoscale Au@GO-zwitterionic chitosan (ZC) particles by efficient modification of the ZC surface without significant changes in size and shape using a single-pass route in a serial gas-phase reactor, and explores potential functions, such as *in vitro* cytotoxicity and inflammation response, for use in biomedical systems. Unmodified chitosan (Cs) (Mw: 15,000 Da, degree of deacetylation: 87%, Polysciences, US) is generally insoluble in water and almost insoluble in organic liquids, so it is challenging to prepare chitosan-based hybrid materials^18^. Thus, ZC, consisting of chitosan and succinic anhydride, is chosen in this work since it has recently been highlighted as one of the modified chitosans that are soluble at neutral pH and can be employed as polymeric carriers^19^,^20^,^21^. Due to unique suppression of inflammatory responses, zwitterionic polymers have recently been employed as a carrier/vehicle of drug delivery systems for use in parenteral applications^22^. As shown in Fig. 1, spark-produced Au nanoparticles were first injected into a collison atomizer filled with GO to form Au@GO nanoflakes, and the nanoflakes were successively injected into the other collison atomizer filled with ZC solution where they were incorporated with ZC precursors to form Au@GO-ZC droplets. The droplets were then thermally cured in an electrically heated tube furnace at a 90 °C wall temperature to extract solvent from the droplets, resulting in Au@GO-ZC hybrid nanoparticles. The particles were thermophoretically collected to expose them to visible light to modify their chemical and surface properties, and finally they were applied to *in vitro* cytotoxicity testing in HeLa cells.

## Results and Discussion

Figure 2 shows the size distributions of Au@GO-ZC nanoparticles compared to individual Au, GO, Au@GO, and ZC particles. The distribution was analyzed using a scanning mobility particle sizer (SMPS, 3936, TSI, US) to determine the mean diameter, standard deviation, and number concentration of the Au@GO-ZC [anhydride to amine ratio (An/Am) = 0.3], which were found to be 174.4 nm, 1.75, and 1.33 × 10^6 ^cm^−3^, respectively. Analogous data for Au@GO and ZC (An/Am = 0.3) were 48.1 nm, 1.66, and 1.87 × 10^6 ^cm^−3^, and 176.5 nm, 1.67, and 1.06 × 10^6 ^cm^−3^, respectively. The results for the Au@GO-ZC were closer to those of the ZC particles than those for the Au@GO flakes. There was no additional peak and only a slight increase in concentration, not in size, suggesting that the Au@GO nanoflakes were well-merged with the ZC, to construct Au@GO-ZC nanoparticles. The other data for “An/Am = 0.7” are described in Table S1 (Supplementary Information) and also demonstrate this incorporation behavior. Moreover, in the case of Au@GO flakes, a nearly quantitative incorporation between Au and GO was also demonstrated. The ZC incorporation intensity *δ* on the Au@GO can be determined using equation^23^, and the results for An/Am = 0.3 and An/Am = 0.3 are 7.8 × 10^−5^ and 8.3 × 10^−5 ^nm^−2^, respectively.


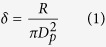


where *R* is the ratio between the number concentrations of ZC and Au@GO flakes, and *D*_p_ is the particle diameter.

Transmission electron microscope (TEM, CM-100, FEI/Philips, US) images (Fig. 3) indicate that the Au particles are agglomerates (consisting of individual Au nanocrystals), while the morphology of the GO particles was hard to define as a certain shape. Nevertheless, the GO particles were well separated. When the Au particles injected into the first atomizer filled with GO flakes, incoming Au particles were located on the GO flakes, forming Au@GO flakes. Interestingly, the Au particles in the form of agglomerates were scattered on the GO flakes in the form of primary particles owing to mechanical restructuring, and their diameter is given by


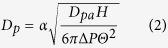


where *D*_p_ is the diameter of a restructured Au, *α* is the proportionality constant, *H* is the Hamaker constant, *ΔP* is the pressure difference, and *Θ* is the cohesive strength parameter. When the Au agglomerates from the spark plasma reactor were injected into the collison atomizer containing an orifice (0.3 mm diameter), and the agglomerates were subjected to different physical conditions (pressure, density, and velocity), the agglomerates shattered. This may further affect no significant differences in particle size distribution between the ZC and Au@GO-ZC cases. For the ZC, the particles had a gradation (dark core-dense solid, bright shell-light solid), which was caused by the given drying rate and can be explained with the Peclet number, *Pe*, which is a dimensionless number that represents the relative time-scales for diffusion (*D*_d_^2^/4*δ*_v_) and convective drying (*τ*_d_).


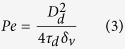


**Figure 1:**
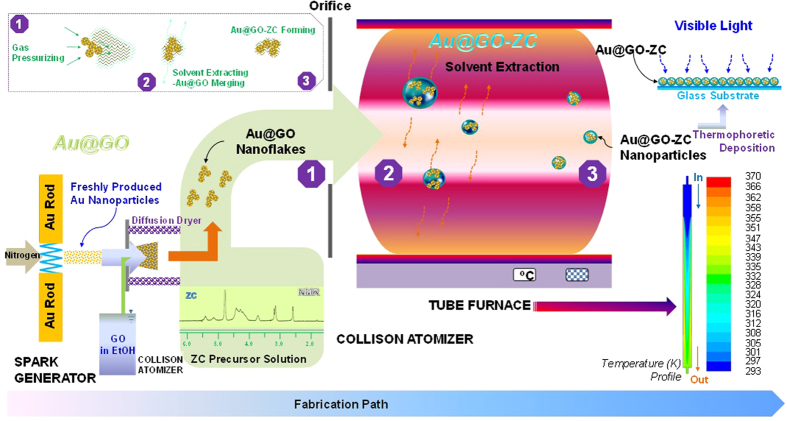
Schematic illustration of the aerosol-based method to fabricate Au@GO-ZC core-shell nanoparticles using a series connection of a spark generator and two collison atomizers.

**Figure 2:**
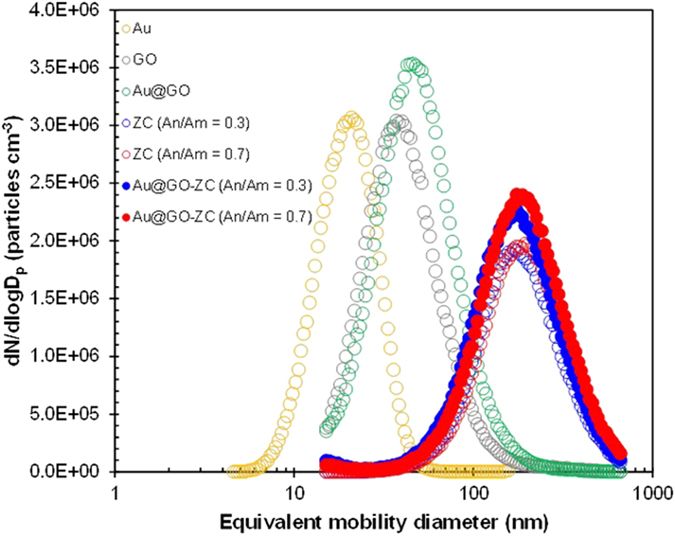
Size distributions of Au@GO-ZC particles in comparison to Au, GO, Au@GO, and ZC particles.

**Figure 3:**
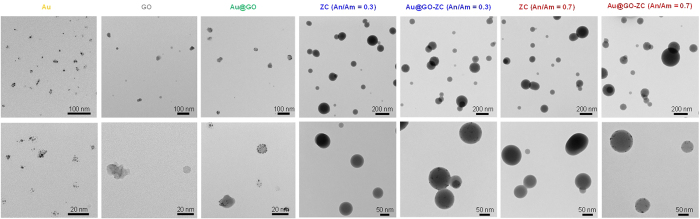
TEM images of Au@GO-ZC particles in comparison to Au, GO, Au@GO, and ZC particles.

**Figure 4:**
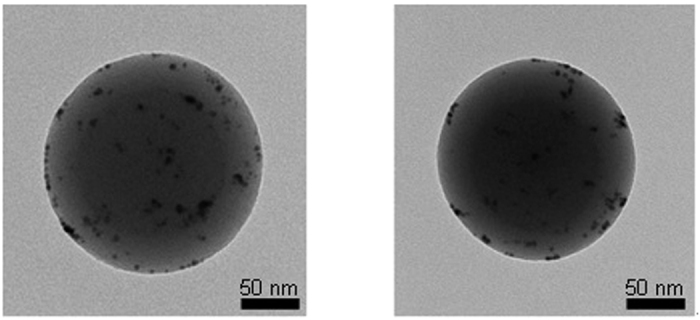
High magnification TEM images of Au@GO-ZC (left-An/Am = 0.3, right-An/Am = 0.7) particles.

The *Pe* value of the current condition was significantly smaller than 1, which introduced the migration of solutes at the interface towards the core region of droplets and was sufficient to keep with the convective drying, thereby inducing the formation of dense solid particles. When the Au@GO flakes were injected into the second atomizer (refer Fig. 1,), the Au@GO particles were incorporated by the ZC droplets owing to the pressurization of the gas. In the case of Au@GO-ZC particles, the high-magnification TEM image (Fig. 4) showed the gray network around Au@GO flakes, implying the presence of a ZC moiety that completely incorporated the Au@GO flakes. The efficiencies of the ZC incorporation of Au@GO flakes for An/Am values of 0.3 and 0.7 were 96.8% and 95.7%, respectively. The mean mode diameters of the pure Au@GO and ZC were 52 ± 4.2 and 171 ± 9.3 nm, respectively. The analogous result for the Au@GO-ZC particles was 176 ± 10.3 nm, which is in good agreement with the results described in Fig. 2 and Table S1 (Supplementary Information). Additionally, the polydispersity index (PDI) value of ZC particles measured by a zetasizer (ZS 90, Malvern Instruments, UK) was 0.286 while that of Au@GO-ZC particles was 0.309, showing suitable particle size distributions (PDI < 0.5).

**Figure 5:**
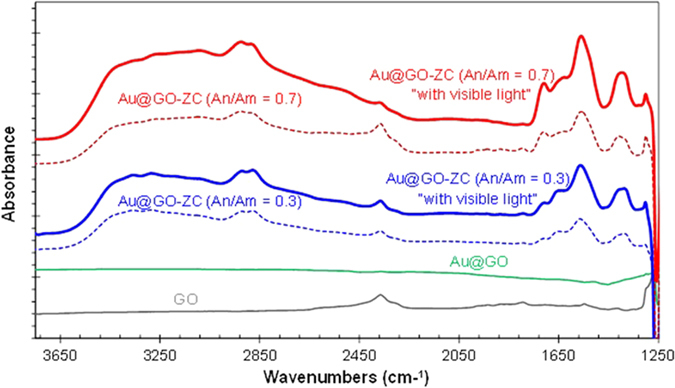
FTIR spectra of Au@GO-ZC particles and GO and Au@GO flakes.

Figure 5 shows the IR profiles of the GO, Au@GO, and Au@GO-ZC particles. The GO spectrum has a peak at 1,730 cm^−1^ (C=O) from carbonyl and carboxylic groups, which supports the existence of oxygen-containing groups (OCGs). The peak at 1,625 cm^−1^ in GO verifies the existence of *sp*^2^ hybridization. When the GO was incorporated with Au, the band for GO disappeared, which implies that nearly all GO particles were quantitatively covered by Au. The Au@GO-ZC exhibited a prominent band at 1,540 cm^−1^, which was attributed to the bending vibration of N-H (amide II) owing to the existence of succinyl groups (*N*-succinylation)^24^. The band for the secondary amide shifted from 1,540 to 1,570 cm^−1^ when exposed to light, indicating the existence of amide bonds between GO and ZC^8^. The spectrum also exhibits the typical absorption bands at 3,360, 2,920, 2,880, 1,680, and 1,360 cm^−1^, which represents the -OH, -CH_2_, -CH_3_, amide I, and amine III groups of pure chitosan, respectively^25^. The relative intensities of characteristic peaks were changed after visible light exposure, especially in the case at An/Am = 0.7 (degrees of modification for An/Am = 0.3 and An/Am = 0.7 are 5.8% and 12.3%, respectively). These results suggest that the photocatalytic degradation process of the pyranose rings occurred with the formation of the carbonyl groups^26^. Considering the work functions of Au (−4.75 eV), GO (−5.35 eV), and ZC (−5.87 eV), electron liberation from ZC to Au@GO is thermodynamically favorable^27^. The ZC is excited first, and subsequently an electron is transferred from the ZC to GO. The liberated electron then moves to an Au particle and is collected by the oxygen to generate reactive OCGs. Eventually, the ZC modifies itself and/or is modified by the reactive species. Moreover, the reduction of the GO is probably owing to a reaction (Fig. S1, Supplementary Information)^28^ between the GO and the amino/hydroxyl groups originated from ZC that may also affect the intensification, because the OCGs of the GO can conjugate with the amino and hydroxyl groups of ZC by bipolar interaction and hydrogen bonding^29^. Figure S2 (XPS spectra, Supplementary Information) further shows the C1s spectra of GO samples with and without visible light exposure. Before the light exposure, the C1s signals deconvoluted into several binding signals for C-C (284.5 eV), C-O (286.2 ev), C = O (287.8 eV), O-C = O (288.9 eV), and O-CO-O (290.8 eV). The fraction of C-C bond (*sp*^2^ carbon) increased from 0.664 to 0.832 via the exposure.

**Figure 6:**
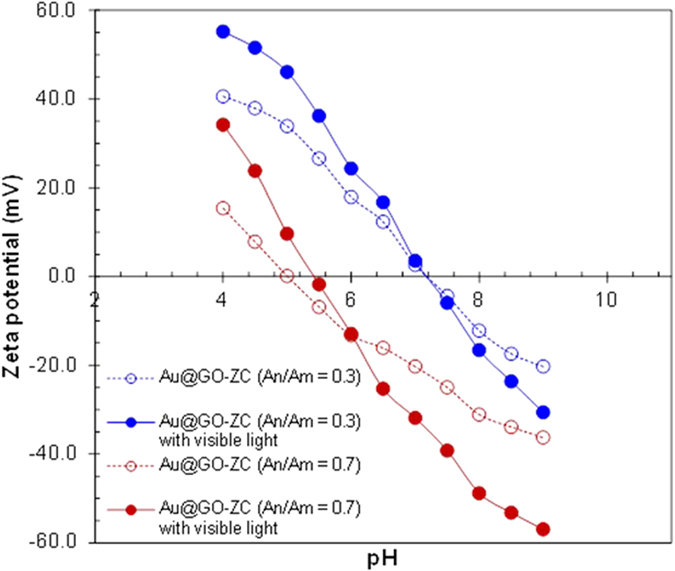
Zeta potential of Au@GO-ZC particles.

The zeta potentials of the Au@GO-ZC particles were measured at different pH values. The particles have positive polarity at acidic pH and negative polarity at basic pH (Fig. 6), which reflects the ongoing deprotonation of the ZC branches. The isoelectric point of the particles changed from 7.2 to 5.3 with the An/Am increasing from 0.3 to 0.7. The polarity was not changed significantly, but the polar component on the surface increased by light exposure. The increase in the polarity of the particles indicated efficient photocatalytic degradation on the surface as noted in Fig. 5, and this may introduce the generation of new polar groups and changes in the microscopic structure via recombination of the polymer. ZC macroradicals formed by the Au@GO flakes during the visible light irradiation may induce the recombination via interaction between the macroradicals. According to negative charges of the particles at pH 7.4 including large sizes (~190 nm), modulations of size and surface area are in progress for efficient drug/gene delivery applications without increases in toxicological/inflammatory responses.

**Figure 7:**
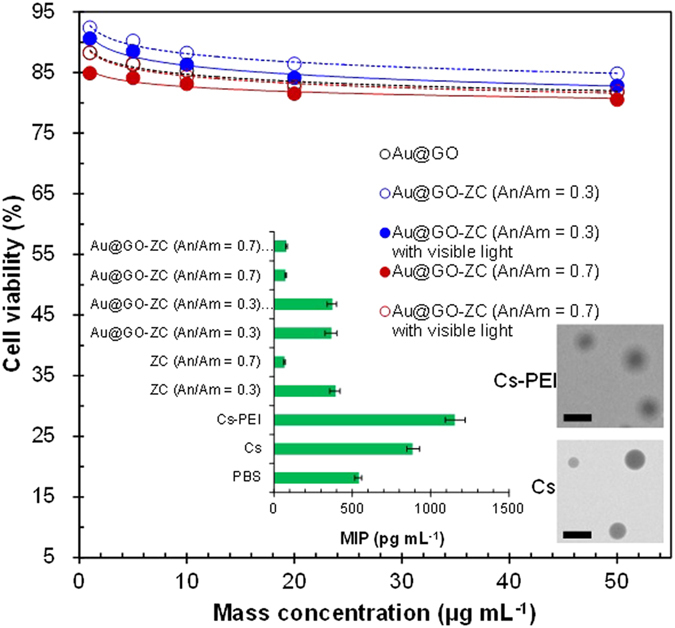
*In vitro* cytotoxicities of Au@GO-ZC particles in comparison to Au@GO flakes. Inset shows the MIP production from LPS-challenged macrophages by adding Au@GO-ZC particles in comparison to ZC particles. Insets also show representative TEM images (scale bar, 200 nm) of the Cs and Cs-PEI particles.

**Figure 8:**
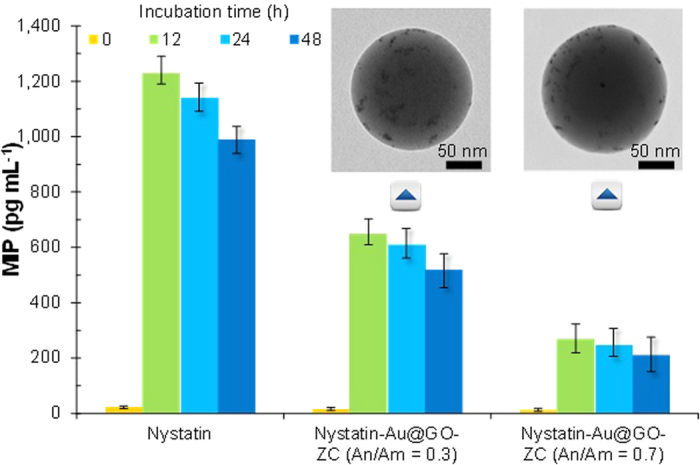
Effect of timed application of Au@GO-ZC particles with nystatin (i.e., nystatin + 10 μg mL^−1^ Au@GO-ZC) on MIP production in the LPS-challenged macrophages. Insets also show representative TEM images of the nystatin incorporated Au@GO-ZC (An/Am = 0.3 and 0.7) particles.

The cytotoxicity of the particles at concentrations of 1, 5, 10, 20, 50 μg mL^−1^ was evaluated by MTS assay in HeLa cells, in comparison to the cytotoxicity of Au@GO (Fig. 7). In order to prepare the particle solution, the sampled particles on a hydrophobic substrate (i.e., polytetrafluoroethylene substrate) were detached by dipping the substrate in water for 10 s in the presence of ultrasound. The measurement results reveal that the cell viability was >80% for the Au@GO-ZC particles even with visible light, while the measured cell viability of the Au@GO flakes was >82%. These results imply that the fabricated Au@GO-ZC particles are biocompatible and non-cytotoxic, and moreover, there were no remarkable differences of cell viability between the cases with and without visible light exposure. The cytotoxicity of the Au@GO-ZC particles (An/Am = 0.3, 0.7) at concentrations at 20–1,000 μg mL^−1^ was also evaluated in murine fibroblasts (L929) as normal cells (Fig. S3). It was observed that the particles did not exhibit higher toxicities even at higher particle concentrations (>100 μg mL^−1^). This implies that the particles warrant further investigation. Furthermore, another scenario for parenteral applications was considered, where Au@GO-ZC particles are administered to tissues that attract activated macrophages. The results (inset of Fig. 7) show that ZC-based particles could more significantly suppress the macrophage inflammatory protein (MIP) production from lipopolysaccharide (LPS)-challenged macrophages than those from Cs and phosphate buffered saline (PBS). This may be due to binding between the ZC surfaces and the cell surface receptors and/or the LPS^30^ that regulate MIP production. The smaller MIP productions of ZC (An/Am = 0.7)-based particles than ZC (An/Am = 0.3) and Cs (also prepared in the gas-phase, inset shows a representative TEM image of Cs nanoparticles) particles indicate that the tendency may be related to the amine content. In addition, polyethyleneimine (PEI) incorporated chitosan nanoparticle^31^ was applied to verify MIP production regarding amine contents that has more amine groups than the Cs, and the results satisfied the hypothesis. There also were no significant increases in MIP production between the Au@GO-ZC particles with and without visible light exposure. The Au@GO-ZC particles were further employed on MIP production (Fig. 8) in the presence of nystatin (10 μg mL^−1^) (N6261, Sigma-Aldrich, US) to highlight another potential of the particles, since nystatin is clinically important as an antibiotic agent^32^. The MIP production reached about 1,250 pg mL^−1^ in the pure nystatin case when the macrophages were incubated for 12 h, and showed an decrease thereafter. The Au@GO-ZC incorporation with nystatin led to significant decreases in MIP production by 520 and 212 pg mL^−1^ for An/Am = 0.3 and An/Am = 0.7, respectively. This might be due to binding cell surface receptors by ZC^33^ that regulates MIP production by nystatin through modifying cell signaling pathways. Insets of Fig. 8 also show TEM images of nystatin incorporated Au@GO-ZC particles (174 ± 6.6 nm for An/Am = 0.3, 179 ± 9.3 nm for An/Am = 0.7), and there are no significant changes in morphology and size, compared with non-incorporated particles (Fig. 4). From the results, the proposed processing method may further be employed to prepare antibiotic particles with anti-inflammatory characteristics for the primary biomedical applications.

For the first time, the modification of ZC was performed by Au@GO nanoflakes under visible light, and the particles were tested for cytotoxicity and MIP production. The visible-light-exposed particles showed different chemical and surface properties from the unexposed cases while there were no significant differences in biocompatibility and increases in MIP production. This work suggests that incorporating fully nanoscale hybrid GO flakes with biofunctional polymers in the gas-phase is a suitable technique for modulating the surface properties of polymers without significant changes in size and shape. These results further establish continuous single-pass processing as an efficient, green, and versatile manner that would be generalizable to design and fabricate a wide range of tunable nanocarriers or nanoscaffolds for both biomedical and scientific purposes. A further study to optimize the proposed method for realistic applications regarding drug delivery systems is now in preparation for publication elsewhere.

## Additional Information

**How to cite this article**: Byeon, J. H. and Park, J. H. Easy on-demand single-pass self-assembly and modification to fabricate gold@graphene-based anti-inflammatory nanoplatforms. *Sci. Rep.* **6**, 34890; doi: 10.1038/srep34890 (2016).

## Supplementary Material

Supplementary Information
