# Supplementary material for: Easy on-demand single-pass self-assembly and modification to fabricate gold@graphene-based anti-inflammatory nanoplatforms
Source: Sci Rep. 2016 Oct 6;6:34890. doi: 10.1038/srep34890 (PMC5052573; doi:10.1038/srep34890)
Supplement: Supplementary Information [file srep34890-s1.pdf]

Supplementary Information for Publication

Easy on-demand single-pass self-assembly and modification to fabricate gold@graphene-based anti-inflammatory nanoplaforms

Jeong Hoon Byeon[1] *& Jae Hong Park*[2]

[1]School of Mechanical Engineering, Yeungnam University, Gyeongsan 38541, Republic of Korea

[2]School of Health Sciences, Purdue University, IN 47907, United States

Correspondence and requests for materials should be addressed to J.H.B (email: postjb@yu.ac.kr) or J.H.P. (email: park895@purdue.edu)

METHODS

- *Fabrication*

Graphite was obtained from a spark discharge system.[S1] GO was prepared from the graphite by using a modified Hummer's method.[S2] Schematic diagrams of the aerosol-based method used for these experiments are shown in **Fig. 1** Freshly spark generated Au was first passed over a collison atomizer containing GO to form AuGO nanoparticles. A pure nitrogen (99.999% purity) flow, which was controlled by a mass flow controller (3810DS, Kofloc, Japan), had a flow rate of 3 L min^{-1} to carry particles. After passed through a diffusion dryer, the AuGO-laden flow was directly employed to further atomize the ZC precursor solution. The ZC precursor solution was used from a method of Xu *et al.*[S3] The AuGO-ZC hybrid droplets then passed through a heated tubular reactor with a 90°C wall temperature for the solvent extraction of the droplets. The condition for complete evaporation can be estimated by considering the time required for the evaporation of the droplets and comparing it with the appropriate residence time in the tubular reactor. The characteristic time to saturate gas with vapor from evaporating droplets, τ, is given *via* the equation,

$$\tau = \frac{1}{2\pi D_d \delta_v C(D_d)} \qquad \text{(S1)}$$

where D_d is the diameter of the droplet, δ_v is the diffusivity of the vapor, and $C(D_d)$ is the droplet number concentration.

- *Instrumentation*

The size distributions of the aerosol particles were measured using a scanning mobility particle sizer (SMPS), consisting of a differential mobility analyzer (3081, TSI, US), electrostatic classifier (3080, TSI, US), condensation particle counter (3776, TSI, US), and a soft X-ray charger (4530, HCT, Korea). The SMPS system, which measured the mobility equivalent diameter, was operated at a sample flow of 0.3 L min^{-1}, a sheath flow of 3.0 L min^{-1}, and a scan time of 135 sec (measurement range: 15.1-661.2 nm). The mass (m) of the fabricated particles was measured using a microbalance (DV215CD, Ohaus, Switzerland) and also confirmed *via* the following equation:

$$m = Q \cdot t_s \int_0^{\infty} \eta(D_p) C_m(D_p) dD_p\ ) \quad \text{(S2)}$$

where Q is the flow rate of nitrogen gas, t_s is the sampling time, $\eta(D_p)$ is the fractional collection efficiency, and $C_m(D_p)$ is the mass concentration of particles.

Transmission electron microscope (TEM, CM-100, FEI/Philips, US) images were obtained at an accelerating voltage range of 46-180 kV. Specimens were prepared for examination in the TEM by direct electrostatic aerosol sampling at a sampling flow of 1.0 L min^{-1} and an operating voltage of 5 kV using a nano particle collector (NPC-10, HCT, Korea).

For Fourier transform infrared (FTIR) spectroscopy analysis, samples were prepared using polytetrafluoroethylene (PTFE) media substrate (0.2 μm pore size, 47 mm diameter, 11807-47-N, Sartorius, Germany) by physical filtration (*i.e.* mechanical filtration mainly by diffusion, of particles on the surfaces of the substrate), and the spectra were recorded on a Nicolet 6700 FTIR spectrometer (Thermo Electron, US). The spectra were taken for samples in the range of 4000-400 cm^{-1} in absorbance mode.

The zeta potential of the fabricated particles was determined using a zeta potential analyzer (Nano ZS-90, Malvern Instruments, UK). Measurements of the zeta potential were carried out at 25°C and calculated using the manufacturer's supplied software.

- *In Vitro Cytotoxicity*

The cytotoxicity of the samples was evaluated using HeLa cells by MTS, 3-(4,5-dimethyl-thiazol-2-yl)-5-(3-carboxymethoxyphenyl)-2-(4-sulfophenyl)2H-tetrazolium, assay. The cells were cultured in 200 mL Dulbecco's modified eagle medium (DMEM, Carlsbad, US) supplemented with 10% fetal bovine serum (FBS) at 37°C, 5% carbon dioxide, and 95% relative humidity. The cells were seeded in a 96-well microtiter plate (Nunc, Germany) at densities of 1×10^5 cells well^{-1}. After 24 h, the culture media were replaced with serum-supplemented culture media containing the sample, and the cells were incubated for 24 h. 30 μL of the MTS reagent was then added to each well. The cells were incubated for an additional 2 h. The absorbance was then measured using a microplate reader (Spectra Plus, TECAN, Switzerland) at a wavelength of 490 nm. The cell viability (%) was compared with that of the untreated control cell in

media without samples and calculated with $[A]_{test}:[A]_{control} \times 100\%$, where $[A]_{test}$ is the absorbance of the wells with samples and $[A]_{control}$ is the absorbance of the control wells. All experiments were performed in triplicate, and the results were reported as means and standard deviations. Statistical analyses were performed using Student's *t*-test. The differences were considered significant for $p < 0.05$.

- *Macrophage Inflammatory Protein Production*

Peritoneal macrophages were seeded in 24-well plates at a density of 10^5 cells per well in 1 mL of medium. After overnight incubation, 0.1 mL of the sample particle solution was injected to each well to set the particle concentraion in medium to 2 mg mL^{-1}. In control groups, 0.1 mL of PBS or unmodified chiotosan was injected in lieu of the sample particle solutions. After 24 h incubation, the culture media were centrifuged at 2000 rpm for 10 min to separate supernatants. Macrophages were challenged by adding LPS to the media in the final concentration of 1 μg mL^{-1} shortly before the Cs and PBS controls. For sample particles, enzyme-linked immunosorbent assay (ELISA) was performed to determine the MIP levels using MIP-2 ELISA kit (R&D Systems, USA). The supernatants collected from LPS-challenged macrophages was always diluted 10 times prior to the analysis. The differences were considered significant for $p < 0.01$.

SUMMARY OF PARTICLE SIZE DISTRIBUTIONS

Case	Mean Diameter (nm)	Standard Deviation (-)	Number Concentration ($\times 10^6$ particles cm^{-3})
Au	19.4	1.46	6.50
GO	41.3	1.65	1.54
Au@GO	48.1	1.66	1.87
ZC (An/Am = 0.3)	176.5	1.67	1.06
Au@GO-ZC (An/Am = 0.3)	174.4	1.75	1.33
ZC (An/Am = 0.7)	189.7	1.69	1.12
Au@GO-ZC (An/Am = 0.7)	186.3	1.78	1.37

FTIR AND XPS SPECTRA OF GO FOR THE CASES BEFORE AND AFTER THE LIGHT EXPOSURE

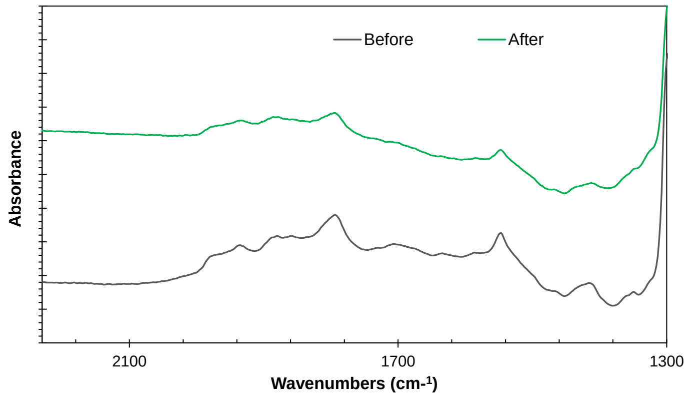

Fig. S1

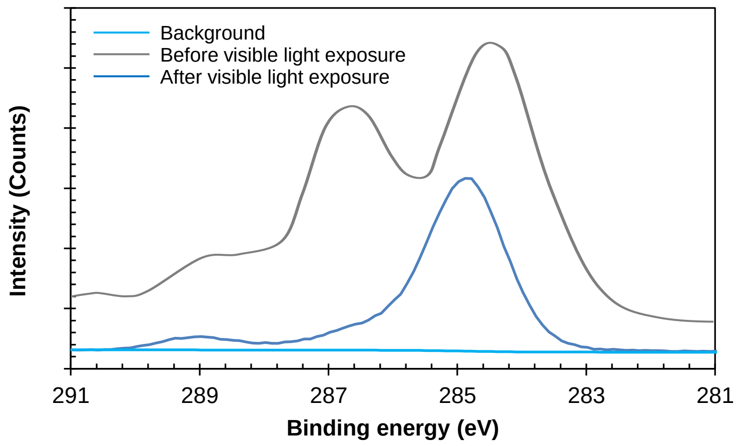

Fig. S2

CYTOTOXICITIES TOWARD L929 CELLS (Green-Live, Red-Dead)

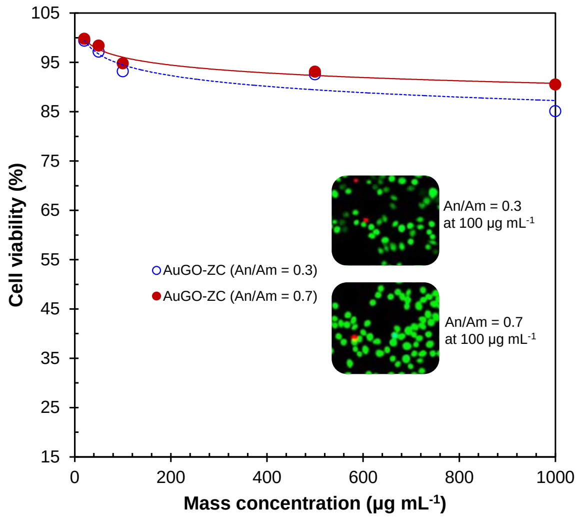

Fig. S3

SUPPLEMENTARY REFERENCES

S1. Byeon, J. H. *et al.* Removal of volatile organic compounds by spark generated carbon aerosol particles. *Carbon* **44**, 2106-2108 (2006).

S2. Hummers, W. S. & Offeman, R. E. Preparation of graphitic oxide, *J. Am. Chem. Soc.* **80**, 1339-1339 (1958).

S3. Xu, P. *et al.* Zwitterionic chitosan derivatives for pH-sensitive stealth coating, *Biomacromolecules* **11**, 2352-2358 (2010).
